# Propensity Score Estimation to Address Calendar Time-Specific Channeling in Comparative Effectiveness Research of Second Generation Antipsychotics

**DOI:** 10.1371/journal.pone.0063973

**Published:** 2013-05-07

**Authors:** Stacie B. Dusetzina, Christina D. Mack, Til Stürmer

**Affiliations:** 1 Division of General Medicine and Clinical Epidemiology, School of Medicine, University of North Carolina at Chapel Hill, Chapel Hill, North Carolina, United States of America; 2 Department of Health Policy and Management, Gillings School of Global Public Health, University of North Carolina at Chapel Hill, Chapel Hill, North Carolina, United States of America; 3 Cecil G. Sheps Center for Health Services Research, University of North Carolina at Chapel Hill, Chapel Hill, North Carolina, United States of America; 4 Department of Epidemiology, Gillings School of Global Public Health, University of North Carolina at Chapel Hill, Chapel Hill, North Carolina, United States of America; Bremen Institute of Preventive Research and Social Medicine, Germany

## Abstract

**Background:**

Channeling occurs when a medication and its potential comparators are selectively prescribed based on differences in underlying patient characteristics. Drug safety advisories can provide new information regarding the relative safety or effectiveness of a drug product which might increase selective prescribing. In particular, when reported adverse effects vary among drugs within a therapeutic class, clinicians may channel patients toward or away from a drug based on the patient's underlying risk for an adverse outcome. If channeling is not identified and appropriately managed it might lead to confounding in observational comparative effectiveness studies.

**Objective:**

To demonstrate channeling among new users of second generation antipsychotics following a Food and Drug Administration safety advisory and to evaluate the impact of channeling on cardiovascular risk estimates over time.

**Data Source:**

Florida Medicaid data from 2001–2006.

**Study Design:**

Retrospective cohort of adults initiating second generation antipsychotics. We used propensity scores to match olanzapine initiators with other second generation antipsychotic initiators. To evaluate channeling away from olanzapine following an FDA safety advisory, we estimated calendar time-specific propensity scores. We compare the performance of these calendar time-specific propensity scores with conventionally-estimated propensity scores on estimates of cardiovascular risk.

**Principal Findings:**

Increased channeling away from olanzapine was evident for some, but not all, cardiovascular risk factors and corresponded with the timing of the FDA advisory. Covariate balance was optimized within period and across all periods when using the calendar time-specific propensity score. Hazard ratio estimates for cardiovascular outcomes did not differ across models (Conventional PS: 0.97, 95%CI: 0.81–3.18 versus calendar time-specific PS: 0.93, 95%CI: 0.77–3.04).

**Conclusions:**

Changes in channeling over time was evident for several covariates but had limited impact on cardiovascular risk estimates, possibly due to unmeasured confounding. Although calendar time-specific propensity scores appear to improve covariate balance, the impact on comparative effectiveness results is limited in this setting.

## Background

Comparative effectiveness research (CER) aims to evaluate the relative benefit or harms of treatment alternatives in patients who are representative of those treated in real-world practice [1], as opposed to the highly selected groups studied in randomized controlled trials. Observational studies of administrative claims are increasingly used for CER of pharmaceutical products. These studies need to address several important sources of bias, including confounding due to changes in the clinical use of treatments over time (i.e., channeling bias) [2]. Channeling occurs when users of a medication and its potential comparators are selectively prescribed a particular agent based on differences in underlying patient characteristics [3]. If these characteristics also affect the risk for the outcome of interest, channeling will lead to confounding.

The impact of calendar time-specific channeling is a particularly important consideration when assessing the comparative safety for drugs within a medication class targeted by a Food and Drug Administration (FDA) safety advisory. In such cases, when reported adverse effects vary among drugs within a therapeutic class, clinicians may respond by channeling patients toward or away from a particular treatment alternative based on the patient's underlying risk for an adverse outcome.

One way to address calendar time-specific channeling is through the use of calendar time-specific propensity scores [4] which estimate the probability of receiving treatment within carefully defined study time periods anchored around changes in policy or new safety or effectiveness information. This propensity score can also provide insight into changes in channeling over time and may provide better confounding control for treatment effect estimates.

### Clinical Context

Second generation antipsychotics (SGAs) are effective medications for the treatment of psychosis, but also have been associated with metabolic adverse effects that increase the risks for cardiovascular morbidity and mortality with long term use [5]. In late 2003, the FDA issued a class-wide advisory regarding increased risks for metabolic adverse effects for patients using second generation antipsychotics [6]. Shortly after the class-wide warning was issued, members of the American Diabetes Association, the American Psychiatric Association, the American Association of Clinical Endocrinologists and the North American Association for the Study of Obesity developed a professional society consensus statement, identifying antipsychotic agents as being of higher or lower metabolic risk based on evidence available at that time [7]. This statement implicated clozapine and olanzapine as the agents with the highest known metabolic risk, with each of the other agents identified as being of either moderate (quetiapine or risperidone) or low/unknown (aripiprazole or ziprasidone) risk.

Previous studies identified large declines in olanzapine use among Medicaid enrollees following the FDA advisory and consensus statement publication [8]–[10]. Additional declines in olanzapine use have been documented in Florida during the years following the advisory, as Florida Medicaid temporarily implemented a prior authorization policy to restrict access to this product in July of 2005 [11]. It is unclear whether these observed decreases in olanzapine use are due to reductions in olanzapine use primarily among patients at higher risk for metabolic adverse events, or if decreases in olanzapine use were non-selective. If the former is true, channeling of patients at higher risk for adverse metabolic-related outcomes away from olanzapine could result in biased estimates of the comparative safety of second generation antipsychotic agents, particularly as related to adverse metabolic or cardiovascular related outcomes.

The objectives of this study are to evaluate (1) changes in channeling of patients away from olanzapine over time; (2) whether covariate balance is improved by matching within calendar time-specific strata; and (3) whether use of calendar time-specific propensity scores results in different cardiovascular risk estimates among second generation antipsychotic users as compared with unadjusted and traditional propensity score matched analyses.

## Study Data and Methods

### Data Source

We used inpatient, outpatient and pharmacy claims from the Florida Medicaid program from 2000–2006 for this analysis. This data source contains inpatient, outpatient and prescription drug utilization data for all paid claims for Florida's fee-for-service Medicaid enrollees. Florida's Medicaid program is the fourth largest in the country and represents an ethnically and racially diverse population [12], [13]. Furthermore, Medicaid insures a large proportion of patients with severe mental illness and paid for the majority of antipsychotic prescriptions in the US during this period [13], [14].

### Study Time Periods

For the calendar time-specific propensity score, we measured incident SGA use during three distinct time segments (January 2001–December 2002; January 2003–December 2003; January 2004–December 2005) that corresponded to relevant publications, media reports, and FDA regulatory activities (See Table S1 for details) [15]–[21].

### Study Design and Cohort Identification

We created a retrospective cohort of new second generation antipsychotic users for our analysis. We selected adults aged 18–64 who were enrolled in Florida's fee-for-service Medicaid program. Enrollees were required to have a new SGA prescription fill between January 1, 2001 and December 31, 2005 and at least 6 months of continuous Medicaid enrollment prior to their index prescription fill date (N = 37,130). SGAs included: olanzapine, quetiapine, risperidone, ziprasidone, and aripiprazole. Clozapine was excluded due to its infrequent use (<1% of SGA fills). From this sample, we excluded individuals with codes for coronary artery disease (acute myocardial infarction, coronary artery revascularization, angina and chronic ischemic heart disease) during the 6 months prior to drug initiation (N = 2,370). Finally, we excluded enrollees who did not fill a second prescription for their index second generation antipsychotic medication within 91 days or those who experienced an outcome of interest prior to their second fill (N = 14,134). This resulted in a final sample of 20,626 Medicaid enrollees.

### Follow Up

We followed these second generation antipsychotic initiators from their second prescription fill date until they experienced a cardiovascular outcome of interest, a gap in their Medicaid enrollment of over 2 months or until December 31, 2006. Enrollees who experienced a change in therapy (switching, augmenting or discontinuing their index prescription (see Table S2 for coding details)) were followed for up to 6 months after their treatment change to identify relevant cardiovascular outcomes allowing for a 6 months carry-over effect.

### Dependent Variable

The primary outcome was coronary artery disease. We defined coronary artery disease as acute myocardial infarction, coronary artery revascularization (either percutaneous coronary intervention or coronary artery bypass grafting), angina or chronic ischemic heart disease (see Table S2 for coding details).

### Covariates

Variables potentially associated with both second generation antipsychotic treatment selection and coronary artery disease, or coronary artery disease alone, were included in the propensity score model. These include: patient age (in years), sex, race (white, black, Hispanic or other), Medicaid eligibility (Supplemental Security Income or other), Medicaid region (categorized as 1–11 based on pre-defined regions within the Florida Medicaid program), prior health services utilization (number of inpatient visits, number of outpatient visits, presence of inpatient services for mental health treatment, receipt of care from a psychiatrist), metabolic or cardiovascular related comorbidities (diabetes, hyperlipidemia, hypertension, obesity, peripheral vascular disease, cerebrovascular disease, cardiac arrhythmias, heart failure), mental health related conditions (schizophrenia, bipolar disorder, psychosis, dementia, substance abuse, major depressive disorder, anxiety disorder), prior medication use indicating or increasing potential for cardiovascular risk (cox-2 inhibitors, NSAIDs, aspirin, antiplatelet medications) and measures of medical comorbidity (using the conditions identified in the Charlson comorbidity index separately) [22]. Variables were dichotomized unless otherwise indicated.

### Analytic Methods–Propensity Score Estimation and Channeling Identification

Propensity scores were estimated using logistic regression by modeling the predicted probability of receiving olanzapine as a function of the covariates measured in the 6 months prior to the index prescription fill date. We created propensity score-matched cohorts in which patients initiating treatment with olanzapine were matched 1∶1 to those initiating any other second generation antipsychotic medication. To create the propensity score matched cohorts we used a 5→1 propensity score digit matching algorithm [23]. In all, there were 47 parameters estimated in the PS model, providing approximately 26 events per predictor using the revised sample/time periods.

To evaluate changes in channeling away from olanzapine over time we estimated propensity scores separately in each pre-defined time period and constructed calendar time-specific propensity score-matched cohorts within each time period. We then combined the resulting datasets for the matched-pairs analysis. To evaluate the relative performance of the calendar time-specific propensity score, we also estimated a conventional propensity score (using all available data from January 2001–December 2005), while controlling for the year of initiation using dummy variables. As with the calendar time-specific propensity score, we used the same pre-treatment covariates in the conventional propensity score model and we utilized the 5→1 digit propensity score matching algorithm to generate matched pairs for analysis. For the conventional propensity score model, matches were made across all periods, rather than restricting to within-period matches.

Adjusted odds ratios from each of the propensity score estimation models were estimated overall and within each calendar time period to identify channeling of patients away from olanzapine over time. Channeling was investigated for covariates that were strong predictors of coronary artery disease and for conditions specifically identified in the metabolic risk advisory and consensus statement. Changes in channeling by covariate over time were assessed using the Cochran-Armitage test for trend. We calculated the absolute standardized mean differences to evaluate between-group covariate balance across key covariates overall and within period to assess the performance of each propensity score matching method [24].

### Analytic Methods–Estimating Model-Specific Changes in Cardiovascular Outcomes

We estimated overall and period-specific hazard ratios for cardiovascular outcomes for patients using olanzapine versus those using other second generation antipsychotics using Cox Proportional Hazard Models. We compare estimates from the unadjusted model with estimates from the conventional propensity score-matched and calendar time-specific propensity score-matched cohorts. Because the propensity score matching techniques are used to balance characteristics of our treatment and control groups we do not include additional covariates in these models.

### Sensitivity Analyses

In sensitivity analyses we reduced the post-censored observation time for enrollees who experienced a change in therapy (switching, augmenting or discontinuing their index prescription) from 6 months to 3 months to determine the extent to which the longer timeframe might bias our estimates of drug-related cardiovascular outcomes. Additionally, as sensitivity analysis for our primary “as treated” analysis, we used an intent-to-treat approach in which individuals were assumed to continue on their index prescription fill until they experienced a cardiovascular outcome of interest, a gap in their Medicaid enrollment of over 2 months or until December 31, 2006.

## Results

### Cohort Characteristics

Before matching there were 7,082 new users of olanzapine and 13,544 new users of other second generation antipsychotic agents included in the sample (Table 1). Of those, 96% of olanzapine users were successfully matched in both the traditional propensity score model and the calendar time-specific model. In the calendar time-specific model, match percentages were 93.4% in period 1, 97.9% in period 2 and 99.8% in period 3. As compared with individuals initiating olanzapine, before matching, those initiating other second generation antipsychotic agents over the study period had less frequent health services utilization (average of 14.6 versus 16.1 outpatient visits in the prior 6 months), and were more likely to have diabetes (11.5% versus 8.5%) and hypertension (27.4% versus 23.9%) and less likely to have pulmonary disease (9.2% versus 11.8%).

**Table 1:** Baseline Characteristics by Sample: 2001–2005.

	*Unmatched Sample*		*Conventional PS Matched*				*TSPS Matched*		
	Olanzapine (N = 7,082)	Other SGAs (N = 13,544)	Olanzapine (N = 6,829)		Other SGAs (N = 6,829)		Olanzapine (N = 6,777)		Other SGAs (N = 6,777)
**Age**									
Age at Index Fill Date – Means (SD)	42.3 (12.2)	41.8 (12.6)	42.2 (12.2)	42.0 (12.5)		42.1 (12.2)		42.2 (12.5)	
18–29	17.9	19.9	18.3	19.1		18.3		18.6	
30–39	21.5	21.2	21.4	21.6		21.5		21.9	
40–49	30.1	28.6	30.9	29.0		30.0		28.3	
50–64	30.5	30.3	30.2	30.3		30.2		31.2	
***Sex***									
Female	57.5	62.1	58.1	58.1		58.0		58.1	
***Race***									
White	46.2	51.7	47.5	47.5		47.9		47.9	
Black	17.1	18.5	17.6	17.8		17.7		17.9	
Hispanic	11.1	8.1	9.8	9.7		9.5		9.4	
Other/Unknown	25.6	21.7	25.1	25.0		25.0		24.9	
***Medicaid Eligibility Category***									
Supplemental Security Income	65.4	64.8	65.9	65.8		66.1		66.1	
**Prior Health Services Utilization – Mean (SD)**									
Number of Outpatient Visits in Prior 6 Months	16.1 (21.2)	14.6 (18.4)	14.5 (18.5)	14.8 (18.7)		14.6 (18.5)		14.7 (18.9)	
Number of Inpatient Visits in Prior 6 Months	1.8 (3.2)	2.0 (3.4)	1.8 (3.2)	1.8 (3.0)		1.8 (3.1)		1.8 (3.2)	
***Metabolic and Cardiovascular Risk Factors***									
Diabetes	8.5	11.5	8.7	8.9		8.7		8.8	
Hyperlipidemia	9.3	9.6	9.0	9.2		8.8		8.4	
Hypertension	23.9	27.4	24.0	23.4		23.9		23.9	
Obesity	1.2	2.1	1.2	1.1		1.2		1.2	
Peripheral Vascular Disease	2.2	1.6	1.9	1.7		1.6		1.8	
Cerebrovascular Disease	3.0	2.9	2.9	2.8		3.0		2.9	

	*Unmatched Sample*		*Conventional PS Matched*		*TSPS Matched*	
	Olanzapine (N = 7,082)	Other SGAs (N = 13,544)	Olanzapine (N = 6,829)	Other SGAs (N = 6,829)	Olanzapine (N = 6,777)	Other SGAs (N = 6,777)
Heart Failure	1.4	1.9	1.4	1.5	1.4	1.5
Pulmonary Disease	11.8	9.2	10.8	10.8	11.2	11.2
Thrombotic Disease	4.8	3.9	4.4	4.4	4.3	4.5
**Other Previous Treatment**						
Aspirin	2.7	3.1	2.6	2.6	2.7	2.6
Non-Steroidal Anti-Inflammatory Agents	14.3	13.2	13.4	13.2	13.4	13.1
Antiplatelet Agents	2.1	2.4	2.1	2.0	2.0	2.1
Cox-2 Inhibitors	13.7	10.9	13.0	12.9	13.0	12.7
**Mental Health Diagnoses**						
Schizophrenia	20.1	19.4	20.4	20.8	20.5	20.4
Bipolar Disorder	10.9	10.5	11.1	10.5	11.0	11.0
Psychosis	2.9	3.8	3.0	2.9	3.1	3.1
**Mental Health-Related Comorbidities**						
Dementia	0.5	0.8	0.4	0.5	0.5	0.5
Major Depressive Disorder	22.6	27.8	23.2	23.3	23.2	23.8
Anxiety Disorder	10.1	11.8	10.3	10.6	10.3	10.3
Substance Abuse	7.8	8.0	7.9	7.8	7.9	7.8
**Prior Psychiatric Treatment**						
Inpatient Mental Health Treatment	21.0	22.8	21.4	21.5	21.4	21.7
Visits to a Psychiatrist	46.2	51.4	47.3	46.6	47.3	47.0
***Other Medical Comorbidities***						
1+	19.4	17.5	18.6	18.5	18.5	18.5
**Incident Prescription Period**						
Period 1 (Jan 2001 – Dec 2002)	57.8	38.0	56.6	56.7	56.3	56.3
Period 2 (Jan 2003 – Dec 2003)	24.7	22.2	25.2	25.0	25.4	25.4
Period 3 (Jan 2004 – Dec 2005)	17.6	39.8	18.2	18.3	18.3	18.3

### Channeling of Patients Away from Olanzapine

We evaluated channeling by period for the top predictors of coronary artery disease in our sample (Table 2). Channeling was not evident for most predictors of coronary artery disease when comparing the odds ratios generated in period 1 to those generated in each subsequent period. However, for patients with prior diagnoses of hyperlipidemia and hypertension we see some evidence that those patients were less likely to receive olanzapine over time (p-value for trend: 0.02 and 0.05, respectively).

**Table 2:** Channeling within and Across Periods for the Top Predictors of Coronary Artery Disease.

Patient Characteristics	Period	Odds Ratio	95% CI	P-value for Trend
Age Over 50 Years	2001-2005	1.07	1.00-1.16	
	Period 1	1.01	0.91-1.12	0.27
	Period 2	1.19	1.03-1.38	
	Period 3	1.07	0.92-1.24	
**White Race**	**2001-2005**	**0.78**	**0.73-0.83**	
	Period 1	0.75	0.69-0.82	0.05
	Period 2	0.72	0.64-0.82	
	Period 3	0.90	0.79-1.03	
**Prior Diabetes**	**2001-2005**	**0.72**	**0.65-0.81**	
	Period 1	0.70	0.60-0.82	0.36
	Period 2	0.73	0.59-0.91	
	Period 3	0.79	0.63-0.98	
**Prior Hyperlipidemia**	**2001-2005**	**1.17**	**1.05-1.30**	
	Period 1	1.28	1.08-1.50	0.02
	Period 2	1.05	0.84-1.31	
	Period 3	0.97	0.79-1.19	
**Prior Hypertension**	**2001-2005**	**0.88**	**0.82-0.95**	
	Period 1	0.95	0.85-1.06	0.05
	Period 2	0.82	0.70-0.95	
	Period 3	0.81	0.69-0.95	
**Prior Heart Failure**	**2001-2005**	**0.73**	**0.56-0.94**	
	Period 1	0.62	0.43-0.88	0.15
	Period 2	0.82	0.48-1.41	
	Period 3	0.94	0.57-1.54	
**Prior Peripheral Vascular Disease**	**2001-2005**	**1.32**	**1.05-1.66**	
	Period 1	1.56	1.14-2.15	0.11
	Period 2	1.12	0.68-1.82	
	Period 3	1.10	0.68-1.78	
**Prior Pulmonary Disease**	**2001-2005**	**1.44**	**1.30-1.60**	
	Period 1	1.28	1.11-1.48	0.08
	Period 2	1.68	1.36-2.07	
	Period 3	1.59	1.29-1.95	

* **P-value is for the interaction between period and the named covariate.**

### Impact of Estimation Strategy on Covariate Balance

We used the average standardized mean difference to assess within- and across-period covariate balance for the top predictors of coronary artery disease (Table 3). Covariate balance was improved in both the conventional and calendar time-specific propensity score matched cohorts as compared with the unmatched cohort. Overall, the calendar time-specific propensity score model produced smaller standardized differences than the conventional propensity score for 23 of 32 comparisons. In the remaining 9 comparisons, differences between the two models were small (usually less than 0.02).

**Table 3:** Covariate Balance within and Across Periods for the Top Predictors of Coronary Artery Disease.

Patient Characteristics	Period	Standardized Difference: Unmatched Cohort	Standardized Difference: Conventional PS	Standardized Difference: CTS PS
Mean Age	2001-2005	0.135	0.054	0.021
	Period 1	0.171	0.084	0.008
	Period 2	0.352	0.112	0.078
	Period 3	0.039	0.118	0.029
**White Race**	**2001-2005**	**0.110**	**0.000**	**0.000**
	Period 1	0.119	0.021	0.010
	Period 2	0.119	0.012	0.012
	Period 3	0.001	0.079	0.014
**Prior Diabetes**	**2001-2005**	**0.101**	**0.009**	**0.005**
	Period 1	0.085	0.004	0.005
	Period 2	0.094	0.040	0.002
	Period 3	0.086	0.021	0.008
**Prior Hyperlipidemia**	**2001-2005**	**0.008**	**0.007**	**0.014**
	Period 1	0.065	0.026	0.011
	Period 2	0.015	0.058	0.014
	Period 3	0.024	0.028	0.023
**Prior Hypertension**	**2001-2005**	**0.082**	**0.012**	**0.001**
	Period 1	0.011	0.034	0.001
	Period 2	0.078	0.040	0.004
	Period 3	0.072	0.021	0.013
**Prior Heart Failure**	**2001-2005**	**0.039**	**0.003**	**0.008**
	Period 1	0.059	0.003	0.019
	Period 2	0.021	0.009	0.005
	Period 3	0.009	0.017	0.019
**Prior Peripheral Vascular Disease**	**2001-2005**	**0.040**	**0.014**	**0.015**
	Period 1	0.065	0.024	0.015
	Period 2	0.022	0.006	0.009
	Period 3	0.005	0.013	0.023
**Prior Pulmonary Disease**	**2001-2005**	**0.086**	**0.003**	**0.000**
	Period 1	0.057	0.020	0.005
	Period 2	0.116	0.003	0.020
	Period 3	0.126	0.076	0.015

### Impact of Estimation Strategy on Hazard Ratio Estimates

We estimated the hazard ratio for coronary artery disease among individuals using olanzapine versus those using any other second generation antipsychotic agent (Table 4). Models were estimated for the entire study period (2001–2005) and separately by period. In unadjusted models we observe a hazard ratio of 0.96 (95% CI: 0.82–3.05) for the full study period. These estimates were unchanged in both the conventional propensity score matched analysis and the calendar time-specific propensity score matched analysis (HR: 0.97, 95%CI: 0.81–3.18, conventional PS; HR: 0.93, 95%CI: 0.77–3.04, CTS PS). Similarly, we found no differences in hazard ratio estimates across any model or any period.

**Table 4:** Hazard Ratio Estimates for Adverse Cardiovascular Outcomes by Model: As Treated Analysis.

Propensity Score Estimation and Application Strategy	Period	Number of Events Olanzapine	Person-Months Olanzapine	Number of Events Other SGA	Person-Months Other SGA	Hazard Ratio	Lower CI	Upper CI
Unadjusted Model	2001–2005	227	105,606	455	203,618	0.96	0.82	3.05
Unadjusted Model	Period 1	158	69,136	190	92,472	1.07	0.86	3.60
Unadjusted Model	Period 2	45	23,516	104	46,432	0.80	0.56	3.16
Unadjusted Model	Period 3	24	12,954	161	64,714	0.74	0.48	3.21

Conventional PS – Matched	2001–2005	214	101,545	232	111,985	0.97	0.81	3.18
Conventional PS – Matched	Period 1	145	65,394	141	70,160	1.05	0.83	3.61
Conventional PS – Matched	Period 2	45	23,197	57	26,203	0.85	0.57	3.48
Conventional PS – Matched	Period 3	24	129,54	34	15,622	0.82	0.48	3.83

CTS PS – Matched	2001–2005	214	100,849	242	109,676	0.93	0.77	3.04
CTS PS – Matched	Period 1	147	64,723	143	67,943	1.04	0.83	3.58
CTS PS – Matched	Period 2	43	23,200	61	26,965	0.76	0.51	3.17
CTS PS – Matched	Period 3	24	12,926	38	14,768	0.72	0.43	3.42

### Sensitivity Analyses

Results from sensitivity analyses reducing post-censored follow-up time from 6 months to 3 months and utilizing an intent-to-treat approach for classifying person-time of exposure were similar to results obtained in the primary analysis (not shown).

## Discussion

Researchers intending to use administrative or secondary data sources for the purposes of comparative effectiveness and safety studies should consider the important role that policies and regulatory decisions play in shaping the use of treatments over time. In prescription drug-related research, the characteristics of populations receiving a particular treatment may vary over time with new drug approvals, new entries into a class, and regulatory warnings regarding emerging safety concerns. Here, we investigate whether concerns about increased metabolic risk for olanzapine shifted prescribing among second generation antipsychotic users.

We found some evidence of diagnosis-specific channeling of patients at higher cardio-metabolic risk away from olanzapine following an FDA advisory and subsequent consensus statement that highlighted these risks. In particular, individuals with prior hyperlipidemia and hypertension became less likely to receive olanzapine over time. However, channeling was not evident for other key risk factors, such as diabetes. Interestingly, in this sample patients with diabetes were less likely to receive olanzapine than other SGAs in each period, even those before the advisory. This suggests that clinicians were selectively prescribing non-olanzapine SGAs for patient with diabetes even before the advisory period.

Conventional propensity score models that control for time provide an overall estimate of the effect of a predictor on the likelihood of using a specific treatment but a “null” effect over a period may represent a higher likelihood of receiving the drug at one point and a lower likelihood of receiving the drug at another. Estimates of the odds of receiving olanzapine among patients with prior hyperlipidemia provide the best demonstration of this from our sample. Here we see that the overall point estimate is 1.17 for the study period, but period-to-period estimates range from 1.28 in period 1 to 0.97 in period 3. Note that the overall estimate is balanced on this measured covariate and would provide a valid estimate over the full study period. However, once we condition on each period (and thus misspecify the propensity score model in at least some of the calendar time strata) covariate balance is no longer achieved using the matched pairs identified in the conventional model.

We had hypothesized that olanzapine use would increase the risk for cardiovascular adverse events prior to the advisory, but that this association would fade over time even with proper control for measured confounders as individuals with higher baseline risk not captured by measured covariates were moved away from olanzapine. In our sample, estimates of the comparative effectiveness of second generation antipsychotics on cardiovascular outcomes did not appear to be influenced by estimation strategy. The small number of outcome events and unmeasured confounding appeared to influence our ability to detect any differences between olanzapine and other SGAs for adverse cardiovascular outcomes.

A key consideration when selecting a propensity scoring strategy is how well the resulting propensity score matched groups are balanced on measured characteristics. While we found only minor differences in the covariate balance between propensity score estimation strategies, we did find that within-period balance was improved by using a calendar time-specific propensity scoring model. While both matching strategies appeared to improve covariate balance overall, investigators who are concerned with investigating within-period differences would benefit from using a calendar time-specific propensity score method. This method is easy to implement and allows for within period comparisons without resulting in “breaking” the matches created in a conventional propensity score model. Additionally, the calendar time-specific propensity score allows appropriate changes in assignment of propensity for treatment receipt for each covariate and forces matches to be made within the investigator-specified time periods so that treatment and control groups are well balanced over each period when changes in channeling may be occurring.

Specific limitations include a lack of information on important unmeasured risk factors for cardiovascular disease (e.g., smoking, family history of cardiovascular disease) or poorly measured cardiovascular risk factors (e.g., potential under-coding of diabetes, hyperlipidemia and obesity) that may influence prescribing of olanzapine. It is important to realize, however, that due to our study design using an active comparator cohort, this only leads to confounding bias if these cardiovascular risk factors also affect channeling between olanzapine and other second generation antipsychotics. While we observed some evidence for bias due to unmeasured confounding, this does not reduce our ability to detect and control for changes in measured channeling over time. Second, the heterogeneous composition of our comparison group may influence our ability to detect differences in channeling and/or cardiovascular risk (e.g., including all non-olanzapine second generation antipsychotic agents, which vary in metabolic risk). Further, as a result of the drug safety advisory, olanzapine use declined steadily over the study period. This reduced the sample size available for later periods. Additionally, other factors may have influenced the use of SGAs over our time period, including drug promotion and drug approvals within the class. Regarding the latter point, a low metabolic risk SGA (aripiprazole) was approved during our study period and may have influenced our estimates of channeling. Next, the best prediction model for detecting channeling may not be the best model to control for confounding, which is the primary goal of the PS model [25]. We implemented the propensity score using matching to simplify the presentation of our results. Propensity score matching allows us to estimate the treatment effect in the treated (olanzapine users). Other researchers may elect to use alternate propensity score implementation strategies (e.g., weighting or stratification), depending on the treatment effect of interest [26]. It will be important for future studies to estimate the impact of these different propensity scoring estimation and implementation methods on both covariate balance and on health outcomes.

## Conclusions

Although calendar time-specific propensity scores appear to improve covariate balance, the impact on comparative effectiveness results is limited in this setting. Future work is needed to examine the utility of this method in observational comparative effectiveness research. Researchers should consider using calendar time-specific propensity scores to improve covariate balance and to identify and potentially reduce channeling bias in studies where prescription drug prescribing practices might have changed over time and calendar time-specific channeling is suspected.

## Supporting Information

Table S1 **Timeframe Selection for Calendar Time-Specific Propensity Score Model.**
(DOCX)

Table S2 **Programmatic Definitions of Select Covariates and Outcome Measures.**
(DOCX)

## References

[1] Sox HC, Greenfield S (2009) Comparative effectiveness research: a report from the Institute of Medicine. Ann oIntern Med 151: 203–205. 10.7326/0003-4819-151-3-200908040-00125 19567618

[2] Hughes MD, Williams PL (2007) Challenges in using observational studies to evaluate adverse effects of treatment. NEJM 356: 1705–1707. 17460224 10.1056/NEJMp078038

[3] Petri H, Urquhart J (1991) Channeling bias in the interpretation of drug effects. Stat Med 10: 577–581. 2057656 10.1002/sim.4780100409

[4] Mack CD, Glynn RJ, Brookhart MA, Carpenter WR, Meyer AM, et al.. (2013) Calendar time-specific propensity scores and comparative effectiveness research for stage III colon cancer chemotherapy. Pharmacoepidemiol Drug Saf. DOI: 10.1002/pds.3386. 10.1002/pds.3386 PMC3659185 23296544

[5] Amiel JM, Mangurian CV, Ganguli R, Newcomer JW (2008) Addressing cardiometabolic risk during treatment with antipsychotic medications. Curr Opin Psychiatry 21: 613–618. 18852570 10.1097/YCO.0b013e328314b74b PMC2678801

[6] F.D.A. U.S. Food and Drug Administration Warning about hyperglycemia and atypical antipsychotic drugs. FDA Safety News, 2004 Available: http://www.accessdata.fda.gov/scripts/cdrh/cfdocs/psn/transcript.cfm?show=28#4. Accessed 2013 Apr 16.

[7] A.D.A (2004) Consensus development conference on antipsychotic drugs and obesity and diabetes. Diabetes Care 27: 596–601. 14747245 10.2337/diacare.27.2.596

[8] Constantine R, Tandon R (2008) Changing trends in pediatric antipsychotic use in Florida's Medicaid program. Psychiatr Serv 59: 1162–1168. 18832502 10.1176/ps.2008.59.10.1162

[9] Morrato EH, Druss B, Hartung DM, Valuck RJ, Allen R, et al (2010) Metabolic testing rates in 3 state Medicaid programs after FDA warnings and ADA/APA recommendations for second-generation antipsychotic drugs. Arch Gen Psych 67: 17–24. 10.1001/archgenpsychiatry.2009.179 20048219

[10] Dusetzina SB, Busch AB, Conti RM, Donohue JM, Alexander GC, et al (2012) Changes in antipsychotic use among patients with severe mental illness after a Food and Drug Administration advisory. Pharmacoepidemiol Drug Saf 21: 1251–1260. 22553074 10.1002/pds.3272 PMC3431449

[11] Signorovitch J, Birnbaum H, Ben-Hamadi R, Yu AP, Kidolezi Y, et al.. (2011) Increased olanzapine discontinuation and health care resource utilization following a Medicaid policy change. J Clin Psychiatry DOI: 10.4088/JCP.09m05868yel. 10.4088/JCP.09m05868yel 21294993

[12] Center for Medicare and Medicaid Services (2007) The Medicaid Analytic Extract Chartbook. Office of Research Development and Information MPR. U.S., Department of Health and Human Services, CMS.

[13] Frank RG, Goldman HH, McGuire TG (2009) Trends in mental health cost growth: an expanded role for management? Health affairs 28: 649–659. 19414870 10.1377/hlthaff.28.3.649

[14] Donohue JM, Huskamp HA, Zuvekas SH (2009) Dual eligibles with mental disorders and Medicare part D: how are they faring? Health Aff 28: 746–759. 10.1377/hlthaff.28.3.746 PMC2773509 19414883

[15] Koro CE, Fedder DO, L'Italien GJ, Weiss S, Magder LS, et al (2002) An assessment of the independent effects of olanzapine and risperidone exposure on the risk of hyperlipidemia in schizophrenic patients. Arch Gen Psych 59: 1021–1026. 10.1001/archpsyc.59.11.1021 12418935

[16] Goode E (2003) 3 Schizophrenia Drugs May Raise Diabetes Risk, Study Says The New York Times. Late Edition – Final ed. New York: The New York Times.

[17] Anand G, Burton TM (2003) New antipsychotics pose a quandry for FDA, doctors. Wall Street Journal. Available: http://online.wsj.com/article/0,SB105002837738499900,00.html. Accessed 2013 Apr 16.

[18] F.D.A. (2004) U.S. Food and Drug Administration, MedWatch Safety Information: Geodon (ziprasidone). MedWatch: The US Food and Drug Administration.

[19] F.D.A. (2005) U.S. Food and Drug Administration, MedWatch Safety Information: Atypical Antipsychotic Drugs.

[20] Lieberman JA, Stroup TS, McEvoy JP, Swartz MS, Rosenheck RA, et al (2005) Effectiveness of antipsychotic drugs in patients with chronic schizophrenia. NEJM 353: 1209–1223. 16172203 10.1056/NEJMoa051688

[21] A.H.C.A. (2005) Florida Agency for Health Care Administration. Agency for Health Care Administration Announces NEW Medicaid Preferred Drug List.

[22] Charlson M, Szatrowski TP, Peterson J, Gold J (1994) Validation of a combined comorbidity index. J Clin Epidemiol 47: 1245–1251. 7722560 10.1016/0895-4356(94)90129-5

[23] Parsons LS (2001) Reducing bias in propensity score matched-pair sample using greedy matching techniques.; 2001 April 22–25, 2001; Long Beach, CA.

[24] Austin PC (2009) Balance diagnostics for comparing the distribution of baseline covariates between treatment groups in propensity-score matched samples. Stat Med 28: 3083–3107. 19757444 10.1002/sim.3697 PMC3472075

[25] Brookhart MA, Schneeweiss S, Rothman KJ, Glynn RJ, Avorn J, et al (2006) Variable selection for propensity score models. Am J Epidemiol 163: 1149–1156. 16624967 10.1093/aje/kwj149 PMC1513192

[26] Ellis AR, Dusetzina SB, Hansen RA, Gaynes BN, Farley JF, et al.. (2012) Investigating differences in treatment effect estimates between propensity score matching and weighting: a demonstration using STARD trial data. Pharmacoepidemiol Drug Saf. DOI: 10.1002/pds.3396. 10.1002/pds.3396 PMC3639482 23280682

