# Supplementary material for: Propensity Score Estimation to Address Calendar Time-Specific Channeling in Comparative Effectiveness Research of Second Generation Antipsychotics
Source: PLoS One. 2013 May 7;8(5):e63973. doi: 10.1371/journal.pone.0063973 (PMC3646952; doi:10.1371/journal.pone.0063973)
Supplement: Table S1 — Timeframe Selection for Calendar Time-Specific Propensity Score Model. (DOCX) [file pone.0063973.s001.docx]

**Table S1: Timeframe Selection for Calendar Time-Specific Propensity Score Model**

| **Period** | **Context and Rationale for Timeframe Selection** |
| --- | --- |
| January 2001- December 2002 | Period prior to publication of metabolic risk information. |
|  |  |
| January 2003 – December 2003 | Period in which peer reviewed publications and media reports began to identify potential metabolic concerns for second generation antipsychotic agents,[^21-23^](#_ENREF_21) but prior to the release of the FDA advisory. |
|  |  |
| January 2004 – December 2005 | Period beginning following the FDA’s advisory and the professional societies’ consensus statement publication[^3^](#_ENREF_3) and mailing of “Dear Health Care Provider” letters.[^24^](#_ENREF_24)^,^[^25^](#_ENREF_25)^,^[^50^](#_ENREF_50) This period also includes a class-wide black-box warning (April, 2005),[^25^](#_ENREF_25) the publication of the Clinical Antipsychotic Trials of Intervention Effectiveness (CATIE) results (September, 2005)[^26^](#_ENREF_26) and the temporary implementation of a preferred drug list in Florida Medicaid (July, 2005).[^27^](#_ENREF_27) The FDA’s black-box warning regarding mortality risk among elderly patients with dementia has been shown to have spillover effects on SGA use for non-elderly patients with FDA-approved indications[^51^](#_ENREF_51) and may decrease overall SGA use in our sample. Next, results from the CATIE trial suggested that, for patients with schizophrenia, olanzapine was the most effective antipsychotic agent studied in terms of discontinuation rates, but was associated with greater weight gain and increases in measures of glucose and lipid metabolism.[^26^](#_ENREF_26) Further, the CATIE trial results suggested that the first generation antipsychotic, perphenazine, was similar to SGAs on measured outcomes.[^26^](#_ENREF_26) These results could potentially lead to an increase in olanzapine use, or an increase in use of first generation antipsychotic agents. Finally, Florida Medicaid announced that, effective July 11, 2005, olanzapine would be reclassified as a non-preferred drug. Per the policy, physicians were given 60 days to switch patients from olanzapine to another agent. However, this policy change was rescinded just prior to the 60 day transition deadline (September 9, 2005) and olanzapine was once again added to the preferred drug list. This policy was previously shown to be effective at reducing the use of olanzapine in Florida Medicaid patients with severe mental illness.[^52^](#_ENREF_52) |
|  |  |
