# Supplementary material for: Propensity Score Estimation to Address Calendar Time-Specific Channeling in Comparative Effectiveness Research of Second Generation Antipsychotics
Source: PLoS One. 2013 May 7;8(5):e63973. doi: 10.1371/journal.pone.0063973 (PMC3646952; doi:10.1371/journal.pone.0063973)
Supplement: Table S2 — Programmatic Definitions of Select Covariates and Outcome Measures. (DOCX) [file pone.0063973.s002.docx]

**Table S2: Programmatic Definitions of Select Covariates and Outcome Measures**

| **Covariate Description** | **Diagnostic Codes Used to Identify Condition** |
| --- | --- |
| **Medical Conditions Indicating Prior Metabolic Risk** | |
| Diabetes | ICD-9-CM codes 250.x; Prescription for an anti-diabetic agent, GPI 27 |
| Dyslipidemia | ICD-9-CM codes 272.0-272.4; Prescription for a cholesterol-lowering drug, GPI 39 |
| Hypertension | ICD-9-CM codes 401.x – 405.x, 437.2; Prescription for an antihypertensive drug, GPI 33, 34, 36, 37 |
| Obesity | ICD-9-CM code 278.0 |
| **Exclusionary Conditions / Outcome – Coronary Heart Disease** | |
| Heart Failure | ICD-9-CM codes 398.91, 402.01, 402.11, 402.91, 404.01, 404.03, 404.11, 404.13, 404.91, 404.93, 428.x |
| Acute Myocardial Infarction | ICD-9-CM code 410.x |
| Coronary Artery Revascularization | PCI - ICD-9-CM codes 00.66, 36.0x; CPT-4 codes 92980-92982, 92973, 92984, 92995-92996  CABG - ICD-9-CM codes 36.1x, 36.2x, 36.31, 36.32; CPT-4 codes 33510-33514, 33516-33519, 33521-33523, 33533-33536 |
| Unstable Angina | ICD-9-CM code 411 |
| Angina Pectoris | ICD-9-CM codes 413.0-413.9 |
| Chronic Ischemic Heart Disease | ICD-9-CM codes 414.0, 414.8, 414.9 |
| Peripheral Vascular Disease | ICD-9-CM codes 440.2, 443.8-444.0, 444.22, 444.81 |
| Cerebrovascular Disease | ICD-9-CM codes 433.x-437.1, 438.x |
| **Mental Health Conditions** | |
| Dementia | ICD-9-CM codes 290.x, 294.1, 331.2 |
| Alcohol or Substance Abuse | ICD-9-CM codes 965.2, 291.1-291.3, 291.5-291.9, 303.0, 303.9, 305.0, 357.5, 425.5, 535.3, 571.0-571.3, 980.x, V11.3, 292.x, 304.x, 305.2-305.9, V65.42 |
| Schizophrenia | ICD-9-CM Codes 295.x |
| Bipolar Disorder | ICD-9-CM Codes 296.0, 296.1, 296.4-296.8 |
| Psychosis | ICD-9-CM codes 293.8, 297.x, 298.x |
| Inpatient Mental Health Treatment | ICD-9-CM between 290.x – 319.x and service type = Inpatient |
| **Censoring Events** | |
| SGA Discontinuation | Discontinuations were defined as 6 or more months of no second generation antipsychotic use following a month of second generation antipsychotic use. |
| SGA Switching | Switching was defined as initiation of a different SGA than was prescribed at the first fill date. |
| SGA Augmentation | Augmentation was defined as initiating a different SGA than was prescribed at the first fill date while remaining on the index SGA. |

GPI = Generic Product Identifier.

ICD-9-CM = International Classification of Diseases, Ninth Revision, Clinical Modification.

CPT = Current Procedural Terminology
